# Genetic Diseases of Fucosylation: Insights from Model Organisms

**DOI:** 10.3390/genes16070800

**Published:** 2025-07-03

**Authors:** Muhammad T. Ameen, Curtis R. French

**Affiliations:** Faculty of Medicine, Department of Biological Sciences, Memorial University of Newfoundland and Labrador, St. John’s, NL A1B 3V6, Canada; mtameen@mun.ca

**Keywords:** fucosylation, LADII, CDG IIC, zebrafish, mouse, drosophila, *C. elegans*, leukocytosis, epilepsy, glaucoma

## Abstract

Fucosylation plays a fundamental role in maintaining cellular functions and biological processes across all animals. As a form of glycosylation, it involves the biochemical addition of fucose, a six-carbon monosaccharide, to biological molecules like lipids, proteins, and glycan chains. This modification is essential for optimizing cellular interactions required for receptor-ligand binding, cell adhesion, immune responses, and development. Disruptions in cellular fucose synthesis or in the mechanisms enabling its transfer to other molecules have been linked to human disease. Inherited defects in the fucosylation pathway are rare, with about thirty patients described. Through genome-wide association studies (GWAS), variants in fucosylation pathway genes have been associated with complex diseases such as glaucoma and stroke, and somatic mutations are often found in cancers. Recent studies have applied targeted genetic animal models to elucidate the mechanisms through which disruptions in fucosylation contribute to disease pathogenesis and progression. Key focus areas include GDP-fucose synthesis through de novo or salvage pathways, GDP-fucose transport into the *Golgi* and *endoplasmic reticulum* (ER), and its transfer by fucosyltransferases (FUTs) or protein O-fucosyltransferases (POFUTs) onto acceptor molecules. Loss or gain of function fucosylation gene mutations in animal models such as mice, zebrafish, and invertebrates have provided insights into some fucosylation disease pathogenesis. This review aims to bring together these findings, summarizing key insights from existing animal studies to possibly infer fucosylation disease mechanisms in humans.

## 1. Overview of Fucosylation

**Figure 1:**
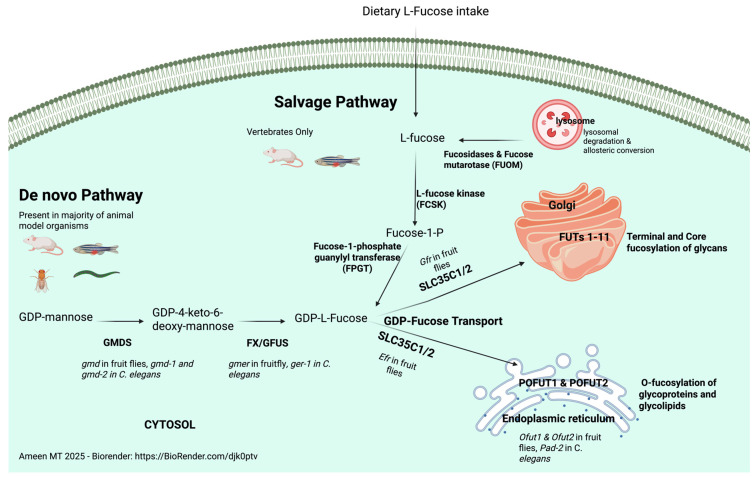
Summary of the main difference in human fucosylation pathways and patterns compared to animal models. GMDS catalyzes the rate-limiting step reaction step for de novo synthesis pathway for GDP-fucose endogenous production in cells, followed by GFUS/FX. The salvage GDP-fucose synthesis pathway is initiated by dietary L-fucose conversion to Fucose 1-phosphate and then to GDP-fucose by FPGT. Salvage pathway GDP-fucose synthesis enzymes have not been identified in invertebrate animal models such as fruit flies and roundworms.

Fucose is a 6-carbon sugar that naturally occurs in the L-Fucose configuration in animals, while other sugars, such as glucose, occur mostly in the D-configuration. Fucose is synthesized in the cell through the de novo synthesis or salvage pathways. The former requires endogenous synthesis of GDP-fucose through a 2-step conversion of GDP-Mannose to GDP-fucose. This is catalyzed by two main enzymes, GDP-Mannose-4,6-dehydratase (GMDS) and GDP-keto-6-deoxy mannose-3,5-epimerase/4-reductase (GFUS/FX protein). 90% of cytosolic GDP-fucose is synthesized through the de novo synthesis pathway [1]. The salvage pathway synthesizes GDP-fucose from free fucose contained in food sources or lysosomal breakdown of recycled glycoproteins and glycolipids. This is a 2-step conversion of L-fucose to GDP-fucose by L-fucose kinase (FCSK) and fucose-1-phosphate guanylyl transferase (FPGT). Cytosolic GDP-fucose is then recognized and transported either to the ER or *Golgi* through transport proteins. SLC35C1 and potentially SLC35C2 proteins initiate GDP-fucose transport to the *Golgi* [2,3,4], and it is not yet clear how GDP-fucose is transported into the ER in humans. Direct glycosylation of glycoproteins and glycolipids likely occurs in the ER, adding fucose directly to polypeptide chains or lipids, while the addition of fucose to other glycans likely occurs in the *Golgi* [5]. The comparison of human fucosylation pathways compared to animal models is summarized in Figure 1.

Nucleophilic addition of GDP-fucose to substrate molecules in the ER or *Golgi* can be in an O-linked or N-linked manner. O-linked fucosylation occurs in the ER by protein fucosyltransferases POFUT1 or POFUT2 that localize to ER membranes [6] and add fucose directly to Serine/Threonine residues on Epidermal Growth Factor-Like (EGF) repeats and Thrombospondin type 1 repeats (TSRs) of target proteins [7,8]. Once fucose has been added to a protein in an O-linked manner, Fringe proteins are required to extend the fucose moiety with additional sugars [9,10]. Other fucosyltransferases (FUTs), eleven of which are identified in humans (FUT1-11), catalyze N-linked transfer of fucose to other sugars, mainly in the *Golgi*. FUT1 and FUT2 catalyze N-linked fucosylation in the lumen of *Golgi*, transferring fucose in an α1,2-linkage to the terminal galactose of lactosamine to make the H antigens on red blood cells [11,12,13]. FUT3-7 and FUT9 add fucose to growing polysaccharide chains via an α1,3/4 linkage and are required for synthesizing Lewis antigen epitopes that comprise the ABO blood group, among others [14,15]. FUT10 and 11, while resembling α1,3 fucosyltransferases, have recently been shown to perform O-fucosylation directly on elastin microfibril interface (EMI) domains and have thus been renamed POFUT3 and POFUT4 [13]. FUT8 is the only known α1,6 fucosyltransferase and is responsible for N-glycan core fucosylation [16,17].

## 2. Diseases of Fucosylation

Disrupted fucosylation, either through defects in GDP-fucose synthesis, transport to the *Golgi*/ER, or transfer to acceptor molecules via FUTs or POFUTs, has been implicated in several human diseases. Congenital disease is rare, encompassing about 30 patients with homozygous mutations and an autosomal recessive mode of inheritance. Variants in fucosylation pathway genes are associated with primary open-angle glaucoma (POAG) [18] and cerebral small vessel disease (CSVD), a stroke risk factor, identified through genome-wide association studies (GWAS) [19]. Altered fucosylation of proteins is also found in tumors, with both increased and decreased fucosylation patterns observed [20].

### 2.1. Congenital Fucosylation Diseases

Given the presence of germline mutations affecting the synthesis, transport, and transfer of the GDP fucose, understanding the symptoms and phenotypes caused by these mutations is of great importance to clinicians, biomedical researchers, and the broader patients’ community. Currently, nineteen patient cases with mutations in *SCL35C1* are reported (OMIM # 266265), who are often diagnosed with leukocyte adhesion deficiency II (LADII) or congenital disease of glycosylation IIc (CDGIIc). These patients all present with developmental and intellectual delay, with the majority having short stature, seizures, feeding problems, and reduced muscle tone. Leukocytosis and recurrent infections are common, with deficiencies in neutrophil rolling due to reduced fucose-mediated binding to endothelial cell selectin proteins [21,22]. Bombay blood group, whereby red blood cells lack the fucose-based A, B, and H antigens, is common in patients with LADII, while cerebral atrophy and ataxic gait were reported in a minority of individuals. Mutations in other fucosylation pathway genes are quite rare. Three patients with fucokinase (*FCSK* OMIM# 618324) mutations have been described with developmental delays, severe infantile-onset epilepsy, and optical abnormalities [22,23,24]. Eight patients with *FUT8* pathogenic variants have also been described (OMIM #618005). Like LAD II, these patients have developmental delay, epilepsy, short stature, and feeding problems [22,25]. One patient presented with congenital glaucoma [26]. A single patient has been described with biallelic mutations in *GFUS* (FX protein, No assigned OMIM#), presenting with global developmental delay, aversion to feeding, and some brain abnormalities upon MRI imaging [27]. No recurrent infections, Bombay blood group, or seizures were recorded for this patient. Homozygous or compound heterozygous mutations in *FUT1* and deletions of *FUT2* have been reported to cause the Bombay blood group in patients cases [28,29,30,31]; however, it is difficult to gauge the number of patients with these mutations, and not all individuals with this rare blood type have been genotyped.

In addition, a heterozygous missense mutation in the *POFUT1* gene is reported to cause the Dowling-Degos disease (DDD) phenotype in multiple Chinese families (OMIM# 615327) as well as one individual from a generalized DDD cohort [32,33]. DDD due to *POFUT1* mutation occurs in an autosomal dominant inheritance pattern and is characterized by hyper- and hypopigmentation of the skin region of the neck, breast, and groin. A single patient harboring a homozygous mutation in *POFUT1* is described as also having global developmental delay, microcephaly, and liver disease [34]. In addition, *GMDS* intragenic deletions have also been directly associated with congenital heart defect with Ebstein anomaly in a 6-year-old girl [35].

Some patients with diseases of fucosylation have been treated with oral fucose or mannose therapy. Such interventions could alleviate disease symptoms in patients with defects in GDP-fucose synthesis but may be limited in efficacy in patients with defects in fucose transport. Of the ~30 patients described with congenital disorders of fucosylation, reports have been generated for at least eleven patients with oral fucose or mannose therapy. Moderate results were obtained, with ten of the eleven patients improving with respect to some disease symptoms and one patient not responding [22,24,36,37,38]. It is therefore important to understand downstream mechanisms resulting from defects in the fucosylation pathways so that additional therapies can be developed.

### 2.2. Fucosylation Association with Complex Disease

While congenital disease of fucosylation is rare, GWAS have identified single nucleotide polymorphisms (SNPs) near or within the *GMDS* gene as being associated with some complex diseases. For example, SNPs within an intron of *GMDS* are associated with white matter hyperintensity volume on T2-weighted MRI images in a large patient cohort from the Framingham Heart Study [19]. Increased white matter hyperintensity volume, an aspect of cerebral small vessel disease (CSVD), increases stroke risk by more than three-fold [39]. SNPs near the *GMDS* gene are also associated with primary open-angle glaucoma [18] and may potentially affect treatment efficacy with latanoprost [40], a common drug for reducing the intraocular pressure. *GMDS*, the gene that catalyzes the rate-limiting step in the de novo synthesis of GDP-fucose, is expressed ubiquitously in the human adult eye [18], indicating a potential role in the maintenance of ocular health. These studies demonstrate that in addition to loss of function mutations that cause congenital disease, smaller changes in the synthesis of GDP-fucose may affect the risk for complex diseases such as stroke and glaucoma. Further research is required to characterize the mechanisms of these congenital and complex diseases of fucosylation using animal models.

### 2.3. Abnormal Fucosylation in Cancers

Disruption to the fucosylation pathway has been reported in different types of cancers. Gain or loss of function mutations or overexpression of genes encoding enzymes in the fucosylation pathway have been reported in cancer animal models and primary cell lines developed from tumors. Aberrant fucosylation could impact cell proliferation, tissue invasion, and metastasis [41]. The ability of cancer cells to spread between tissues depends on their ability to bind to tissue and roll to other locations, and this may be facilitated by the overexpression of fucosylated sialyl Lewis antigen on cancer cells, thus increasing their adhesion and binding capacities [42,43]. Also, disrupted fucosylation has also been reported to cause abnormal immune surveillance evasion of tumors due to loss of *GMDS* and *FUT8* genes [44,45]. Of note, deletion of *GMDS*, the gene required in the first step of GDP-fucose synthesis from mannose-based substrates, is found in up to 13% of colorectal cancers [46]. Changes in core fucosylation facilitated by *FUT8* are associated with poor prognosis and metastasis of many cancers, including hepatocellular carcinomas, breast, colorectal cancer, lung, melanoma, and others [45]. Increased fucosylation of alpha-fetoprotein has also been noted in the sera of patients with early hepatocellular carcinoma (HCC) and germ cell tumors, providing an early marker of tumor formation and metastasis and reoccurrence after curative treatment [47,48]. Additionally, up-regulation of *GMDS* mRNA and protein has been shown in lung adenocarcinoma compared to surrounding normal tissue [49]. Dysregulated expression of *FUT1* has been reported in primary tumors and cancer cell lines, including bladder, pancreatic, hepatocellular, colorectal, breast, oral, head/neck, melanoma, prostate, and cervical cancers [20,50,51]. The removal of fucose from proteins, which occurs in the lysosome, may also be implicated in cancer. The removal of fucose via FUC1 is under transcriptional control of the p53 tumor suppressor protein, linking the breakdown of fucose-containing proteins to cancer progression [52,53].

### 2.4. Fucosylation of Notch Receptors and Disease

Specific mutations in the genes that alter the fucosylation of EGF repeats on Notch receptors have also been implicated in human disease. Notch receptor extracellular domains contain EGF-like repeats that require fucosylation by POFUT1 in the endoplasmic reticulum. Mutations in the *NOTCH3* receptor gene are implicated in CADASIL (cerebral autosomal dominant arteriopathy with subcortical infarcts and leukoencephalopathy) due to mutations in the EGF-like repeats of the NOTCH3 receptor extracellular domain [54]. In such cases, extension of fucose glycans by Fringe proteins is impaired [55]. Similarly, mutations that affect NOTCH receptor extracellular domain EGF repeats or their ability to extend O-linked fucose on such repeats have been implicated in skeletal and muscular diseases such as congenital scoliosis (CS) and spondylocostal dysostoses (SCD). These skeletal diseases are caused by vertebral malalignment during development, thus leading to abnormal thoracic cage and rib numbers. Mutations in the NOTCH ligand *DLL3* (OMIM# 277300) and *LUNATIC FRINGE* (*LNFG*) can cause these skeletal diseases in an autosomal recessive manner [56,57,58].

## 3. Animal Models of Fucosylation Disease

Animal-based models of fucosylation diseases have been generated to understand the basic underlying mechanisms exerted by abnormal fucosylation in human disease progression. Such reports from mice, rats, zebrafish, fruit flies, and roundworms have confirmed the evolutionary role of the fucosylation pathway in embryonic development and its role in congenital disease and support the known associations of fucosylation pathway variants with complex disease risk. Although other animal models such as mice can be explored to investigate a combination of germline congenital disorders and complex diseases caused by disrupted fucosylation genes, given the current advocacy and support for a 3Rs approach in animal research, zebrafish or invertebrate models may be more appropriate to understand disease mechanisms that may provide drug targets for therapeutic development. This section will explore available animal model studies of fucosylation disease, summarizing the key findings from these works. Table 1 illustrates the published animal models for fucosylated-related diseases.

### 3.1. Mouse and Rat Models of Fucosylation-Related Diseases

The mouse, *Mus musculus*, is a useful animal model to study human disease mechanisms and for the development of therapeutics. Due to their genetic proximity to humans, mice are the most used model organism for human disease biomedical research [73]. The mouse genome is 14% smaller than the human genome, with over 90% of the mouse genes having evolutionary conserved homologs in the human genome [74]. Mice also utilize the de novo and salvage pathways for GDP-fucose synthesis.

A mouse model for disorders of fucosylation was produced that harbors a null mutation in *Gfus* (Fx protein) via targeted locus knockout. Progressive intrauterine loss of homozygotes was noted, with live births of homozygotes thus occurring at less than Mendelian ratios [65], reflective of the rarity of *GFUS*-based disease (one known individual). Leukocytosis, accounted for by a 25-fold increase in neutrophils, occurred in these mice. While most patients with congenital disorders of fucosylation display such phenotypes [22], white blood cell counts were not altered in the single patient described with *GFUS* mutations. This discrepancy may result from the nature of mutations, with the patient harboring two different predicted pathogenic variants [27] versus the mouse with a homozygous deletion of the *Gfus* locus.

*Gfus*^-/-^ mice also display adenocarcinoma and colitis, diseases often associated with fucose deficiency. Furthermore, loss of *Fut2* in mice leads to similar phenotypes, implying that the addition of fucose via α1,2-linkage is important for the health of intestinal mucosa [75]. Defective Notch signaling was implicated in the development of these intestinal phenotypes, as loss of the Notch target gene *Hes1* was observed [66]. A separate study examining *Gfus*^-/-^ mice noted goblet cell hyperplasia and growth retardation on fucose-free diets. This phenotype was lessened via reestablishment of Notch signaling, again resembling congenital disease of fucosylation and colon cancer-like phenotypes [67]. Addition of fucose to the diet reduced tumor formation and the leukocytosis in the *Gfus*^-/-^ mouse model, demonstrating that the salvage pathway offers a viable treatment against such disease phenotypes. Notably, the single patient with *GFUS*-based disease also improved with fucose-based therapy.

While there are no reports of a mouse harboring *Gmds* mutations, a xenograft mouse model was developed to assess the role of *GMDS* in tumors, given that *GMDS* mRNA is often upregulated in lung adenocarcinoma patients. Nude mice were transplanted with lung adenocarcinoma H1299 cells, and cell groups were either infected with scrambled-shRNA or GMDS-shRNA lentivirus particles. Knockdown of *GMDS* reduced tumor size and tumor growth when compared to the control group with scrambled-shRNA-infected cells [49]. This study demonstrates that loss of de novo fucosylation, either through global deletion of *GFUS* (*FX*) or through inhibition of *GMDS*, can have profound effects on lung, liver, and colon cancer development in addition to LADII phenotypes such as neutrophilia.

Loss of function mutation of the GDP-fucose transporter gene in mice, *Slc35c1*, required for the transport of GDP-fucose into the Golgi, also causes defects observed in LADII patients. Leukocyte rolling was highly reduced in such mice [59,60], with a subsequent 89% reduction in neutrophils homing to areas of inflammation. These mice also displayed growth retardation, similar to *Gfus*^-/-^ mice and patients harboring mutations in *SLC35C1*. While the related *SLC35C2* has been proposed to facilitate transport of fucose into the ER, double *Slc35c1/Slc35c2* mutant mice were indistinguishable from *Slc35c1* single knockouts. Fucosylation in the ER occurred in the double *Slc35C1/Slc353C2* null mice [4], and knockout of *Slc35C2* did not affect Notch signaling. This indicates that a yet to be discovered ER-specific fucose transporter must exist in mice. Assessment of other phenotypes, including colorectal cancer, stroke, or glaucoma, was not tested in these models; however, additional phenotypes, including dilated lung alveoli and hypocellular lymph nodes, not described in patients, were noted [59].

*Fut8* null mice have also been generated and are born at Mendelian ratios. Although phenotypically indistinguishable from wild-type littermates, most *Fut8*^-/-^ mice die by day 3, and those that survive display significant growth retardation [64,76], similar to *Gfus*^-/-^ and *Scl35C1*^-/-^ mice. Abnormal lung development was noted with emphysema-like phenotypes, owing to dysregulation of TGF-Beta signaling leading to upregulation of MMP proteins. Other phenotypes common in patients with mutations in *FUT8*, such as epilepsy and microcephaly, were not tested in this model. Lastly, conditional knockout of *Pofut1* in mouse endothelial cells facilitated liver injury-induced fibrosis [77], which is noteworthy given a single patient described with liver disease due to homozygous mutation of *POFUT1* [34].

Rat models have also been used to study the fucosylation pathway. While knockout of fucosylation pathway genes has not occurred in rats, researchers used oral supplementation of a fucosylated oligosaccharide (2′-fucosyllactose) in newborn rats, which has been shown to improve cognitive skills later in life and maintain long-lasting increased long-term potentiation [78]. Also, in memory studies of passive avoidance response (PAR), rat models show that transient chemical inhibition of protein fucosylation through intracerebroventricular administration of 2-Deoxy-D-galactose (do-gal, which prevents terminal fucosylation) disrupts memory trace processing. This indicates that fucosylation is required at some phases for memory retention [79] and supports the role of this pathway in regulating cognition and memory, which are concerns for patients with diseases of fucosylation. In addition, in a rat model of renal fibrosis, core fucosylation by *FUT8* is shown to increase the expression of the TGF-beta superfamily receptor, thus causing unilateral ureteral blocking of the kidney. Knockdown of *Fut8* by adenoviral-mediated antisense inhibition in vivo slowed progression of renal fibrosis [80], owing to reduced core fucosylation of transforming growth factor B1 (TGF-β1) and Activin receptor-like kinase 5 (ALK5) receptors.

### 3.2. Zebrafish as a Model for Fucosylation in Development and Disease

The zebrafish, *Danio rerio*, is an excellent animal model for studying cellular development and embryogenesis in metazoans. Notably, 82% of the genes listed as causing morbidity in the Online Mendelian Inherited Disease in Man Database (OMIM) have at least one ortholog in the zebrafish genome, and 76% of genes associated with complex disease using GWAS have at least one ortholog in the zebrafish genome [81]. With their optical clarity, rapid development, and the ease of creating loss of function mutations in vivo, zebrafish allow for direct visualization of molecular changes during development.

Given the reported association of SNPs in an intron of human *GMDS* with stroke risk [19], Fowler et al., 2021 [69], used zebrafish to study the effect of fucosylation loss in hemorrhagic stroke. In this study, a loss of function mutation in the *gmds* gene was created using CRISPR/CAS9 gene editing. This caused an early-onset cerebral hemorrhage phenotype starting from 2 days post fertilization (2 dpf). Also, smooth muscle cell recruitment onto the nascent vasculature was reduced at 2 dpf in *gmds* mutants around the pharyngeal arch arteries and the heart. Ectopic endothelial cell branching was observed. Notch signaling was shown to regulate the *gmds* loss of function-induced cerebral hemorrhage, as activated Notch signaling through overexpression of the Notch Intracellular Domain (NICD) rescued hemorrhage frequency in the homozygous *gmds* mutants. These findings further underscore the importance of fucosylation and Notch signaling in vascular disease development, such as in stroke and other cerebral vascular anomalies. Zebrafish have a fucosylation salvage pathway, as elegantly demonstrated through the feeding of labeled fucose analogs to zebrafish [82]. Injection of L-fucose into the zebrafish yolk sac at early stages could partially rescue the hemorrhage frequency in *gmds* mutants [69], demonstrating the utility of the salvage pathway in the treatment of disease resulting from defects in GDP-fucose synthesis.

It has recently been shown that *gmds* loss of function affects glaucoma development using zebrafish. Glaucoma is caused by progressive optic nerve damage and retinal ganglion cell (RGC) loss and is the leading cause of permanent blindness worldwide, with Primary Open Angle Glaucoma (POAG) being the major subtype. Common clinical findings in POAG include increased intraocular pressure that damages the optic nerve, leading to loss of RGCs, although other mechanisms have been proposed. Optic nerve damage and RGC loss were assessed in aging *gmds* heterozygous zebrafish using optical coherent tomography (OCT) imaging and histology [70]. RGC loss and optic nerve head damage were observed in these fish. Transcriptome sequencing of the eye tissue also revealed a significant downregulation of *crystallin* genes in the *gmds* heterozygotes [70]. Crystallin proteins provide a stress response function to prevent the aggregation of misfolded proteins in the lens and retina [83,84] and have been associated with glaucoma and RGC loss in some patients [85] and in vitro models [86,87]. Thus, findings from zebrafish agree with human GWAS that highlight *GMDS* variants increase the risk of POAG. Mechanistically, this indicates that loss of *GMDS* in humans may influence glaucoma development through a deregulated stress response as the human ages.

Song et al., 2010 [71] also created a zebrafish *slytherin* mutant *(srn)* with missense mutation in *gmds* to study congenital diseases of glycosylation. This mutant allele was isolated from a forward genetic screen for defects in synaptogenesis at the neuromuscular junction, causing abnormal swimming patterns. Abnormal swimming has often been used as a surrogate phenotype for ataxic gait, observed in a minority of patients with disease of fucosylation [22]. The *slytherin* mutants develop a bent tail at 24 hpf that worsens progressively and malformation of the hindbrain at 48 h post fertilization (hpf), which was also observed in the CRISPR-generated INDEL mutant described previously by Fowler et al., 2021 [69]. GDP-fucose supplementation was shown to rescue the *Slytherin* phenotype. While a detailed analysis of the vertebrae has not been undertaken in these animals, it is noteworthy that mutations in Fringe genes, required to elongate polysaccharide chains after the addition of fucose, can cause scoliosis with vertebral fusions [56,57]. Loss of fucosylation in the mutants additionally caused defects in neuronal differentiation and maintenance, highlighting similarities between zebrafish *gmds* mutants and patients with congenital disorders of glycosylation, who often present with intellectual disability and developmental delay. Some of the defects in these mutants were attributed to defects in Notch signaling, others were Notch independent, suggesting that fucosylation of other receptors may affect signaling pathways that regulate neuronal differentiation and function. Another zebrafish missense *gmds* mutant, named *towhead* (*twd*), showed similar phenotypes, showing defects in vagus motor neuron differentiation and cerebral hemorrhage [69,72]. While these three zebrafish *gmds* mutant strains clearly demonstrate overlapping phenotypes with patients with diseases of fucosylation, common phenotypes associated with LADII, such as epileptic seizures and recurrent infections due to defects in neutrophil rolling, have not yet been assessed.

Recently, a novel mutation in the zebrafish *fcsk* gene was reported [62]. Like the two described patients with *FCSK*-attributable disease, these zebrafish displayed developmental delay and neurodevelopmental disorders. Both patients with mutations in *FCSK* were diagnosed with early-onset epilepsy, while the *fcsk* mutant zebrafish displayed increased susceptibility to the convulsant agent Pentylenetetrazol (PTZ) that is often used as a surrogate phenotype for epileptic seizures [88]. Notably, overexpression of the human *FCSK* gene rescued these phenotypes, demonstrating the evolutionary functional conservation of the salvage pathway between teleost fish and humans.

In addition to the generation of mutant strains, antisense inhibition of some fucosylation pathway genes has been used in zebrafish to elucidate gene function and corresponding phenotypes. Zebrafish have been used to study the development of Dowling-Degos disease (DDD), characterized by hyper and hypopigmentation of the skin around the breast, neck, and groin area due to mutations in *POFUT1*. Morpholino knockdown of *pofut1* in zebrafish causes hypopigmentation at 48 hpf and abnormal melanin spread at 72-h post fertilization (hpf) [89]. Tyrosinase activity, required for melanin production, was reduced up to 45%. Reduced expression of Notch ligands and downstream Notch targets was observed, again pointing to the critical role of fucosylation in Notch signaling. Findings from this study elucidate the role of fucosylated glycans in melanin transport and synthesis and the versatility of using zebrafish to study human disease. Additionally, antisense inhibition of *fut8* has been performed in zebrafish, with defects in myogenesis noted [63]. The majority of LADII patients display reduced muscle tone, again underscoring the use of zebrafish to model these rare genetic diseases.

### 3.3. Invertebrate Models of Fucosylation Disease

*Drosophila melanogaster*, the common fruit fly, has been used to model human disease, as has the free-living worm, *Caenorhabditis elegans*. Both organisms have been instrumental in assaying phenotypes and mechanisms. Homologs of human de novo fucosylation pathway genes have been described in *Drosophila* and *C. elegans*, although neither invertebrate displays evidence of a salvage pathway [90,91].

*Drosophila* has a single homolog of the human *GMDS* (*gmd*) and *GFUS* (*gmer*) genes required for the de novo pathway synthesis of GDP-L-fucose. A specific *Golgi* GDP-fucose transporter, termed *Gfr*, is highly similar in sequence to *SLC35C1*, and of note, a specific ER GDP-fucose transporter, *EFr*, has also been defined. While no ER-specific GDP-fucose transporter has been characterized in mammals, experimental evidence clearly points to its existence [4]. Fucosyltransferases, particularly *Ofut1* (previously known as neurotic, *nti*) and *Ofut2*, which catalyze O-linked fucosylation on *Drosophila* EGFRs and thrombospondin type 1 repeats (TSRs), have also been reported [68,92,93]. Additionally, at least four genes encoding enzymes with α1,3-fucosyltransferase activity (FucTA, FucTB, FucTC, and FucTD) are found in the *Drosophila* genome [94], and one α1,6-fucosyltransferase, similar in sequence to human *FUT8*, has been described [95]. No genes encoding α1,2-fucosyltransferase activity are found using mammalian homologs as bait, indicating that this specific linkage does not occur in *Drosophila*, consistent with what has been observed in other insects [95,96].

*Drosophila* has been used to study human congenital disorder of glycosylation using a neuronally altered carbohydrate (*nac*) mutant strain. This strain was shown to have a mutation in the *Golgi*-specific fucose transporter, *Gfr*, causing a nucleotide substitution in the mature protein. The mutants lack terminal α1,3-linked and α1,6-linked fucose residues [97] and display reduced neural fucose-based epitopes as well as Notch-dependant wing tissue loss [61]. With implication to its role in colorectal cancer, GDP-fucose biosynthesis is also important for maintenance of stem cell population in the intestine of *Drosophila*, as *gmd* mutants displayed aberrant, self-renewing stem cell divisions that generated extra stem cells defective in Notch signaling [98]. The *Drosophila* neurotic (*nti*) mutant, with a mutation in the *Drosophila* homolog of *POFUT1*, also displays defects in Notch signaling. Work with this mutant demonstrates that O-fucosylation of EGF repeats on the *Drosophila* Notch receptor is required for ligand binding and the activity of Fringe proteins that extend fucose residues with additional sugar moieties [68]. Also, fruit flies have been used as a model to demonstrate the importance of fucose in regulating susceptibility to *Candida albicans* infections, as an RNAi screen identified downregulated FucTA (an alpha1,3 fucosyltransferase) to cause an increased risk and severity of *C. albicans* infection. [99].

The worm, *C. elegans*, has 2 genes encoding the human *GMDS* gene (*gmd-1* and *gmd-2*) and one gene encoding the human *Gfus* gene homolog (*ger-1*) [68]. A number of fucosyltransferase genes have been cloned, including genes that encode α1,3 fucosyltransferase activity (*fut-1*, *fut-3*, *fut-4*, *fut-5*, and *fut-6*, or CEFT1-5 proteins) [100]. Two genes encoding a fucosyltransferase with α1,2 fucosyltransferase activity had been characterized (*Ce2ft1*, *Ce2ft2*), facilitating the transfer of fucose similar to mammalian FUT2 proteins and are required for the generation of H antigens [101,102]; however, many more are predicted from bioinformatic-based searches [14]. α1,6 fucosyltransferase genes highly similar to human *FUT8* activity have been observed in roundworm species. [14,95]. Also, a *POFUT2* homolog, known as *pad-2* in *C. elegans*, has been shown to be required for the roundworm’s normal development, as shown in *pad-2* mRNA inhibition through RNAi screening and overexpression experiments [103].

*C. elegans* have been used to study *Bacillus thuringiensis* and *Helicobacter pylori* infection. *B. thuringiensis* toxin-resistant (*bre*) mutants were found to have a mutation in the *gmd-1* gene, indicating that fucosylated glycans are important for infection of the roundworm GI tract. Injection of GDP-fucose, but not L-fucose, was able to rescue these phenotypes [90] in agreement with genetic studies that find no salvage pathway genes in the *C. elegans* genome. While the majority of human patients with fucosylation-based disease have increased rates of infection, this has mainly been attributed to the dysfunction of the innate immune response involving reduced neutrophil rolling. Studies in *C. elegans*, which lack neutrophils but display increased susceptibility to infection, highlight the possibility that other mechanisms may also play a role.

The roundworm provides the opportunity to create mutations in genes required for the de novo synthesis and transfer of fucose. While mutations in fucosyltransferase genes such as *fut8*, *fut6*, and *fut1* have been published and demonstrate the resulting changes in glycome biology [104], no phenotypic analysis has been performed. In contrast, a detailed phenotypic description of the disruption of *pad-2* via RNAi or its overexpression has been described, with reduced function of *Pad-2* leading to morphological abnormalities and improperly positioned nerve cords and muscle cells. Increasing the dosage of *Pad-2* caused highly penetrant embryonic lethality, and surviving embryos displayed morphological defects. These studies highlight the evolutionarily conserved role of O-linked fucosylation in muscle and neuronal systems development. While *Pofut* genes have been shown in vertebrate models to affect Notch signaling leading to such phenotypes, an analysis of Notch signaling with knockout or knockdown of *Pad-2* in *C. elegans* has not been undertaken.

## 4. Summary

Animal models of fucosylation and disease pathology have elucidated pathways and interventions that could be targeted to treat human disease. Model organisms, including fruit flies, roundworms, zebrafish, mice, and rats, have significantly advanced our understanding of the biological roles of fucosylation and its disruption in human diseases. Each model contributes unique advantages and limitations, helping us to explore molecular mechanisms, developmental processes, and therapeutic strategies for human diseases. *Drosophila* and *C. elegans* simplify genetic studies and have provided important insights on the role of fucosylation in infection and Notch signaling. Zebrafish provide real-time developmental insights and have demonstrated both Notch-dependant and independent mechanisms for phenotypes associated with defects in fucosylation. Invertebrate models and zebrafish, owing to the high-throughput nature of experimentation, allow for drug screening that may identify compounds that alleviate phenotypes resulting from defects in fucosylation. Such compounds would be beneficial to patients who do not respond to oral fucose therapy, such as subsets of patients with defects in fucose transport or transfer to acceptor molecules that would not be expected to respond to treatment [22,38].

Mice and rats bring behavioral relevance to disease modeling in a vertebrate closely related to humans and have been critical in understanding the role of fucosylation in learning and memory, cancer biology and diseases of the gastrointestinal tract. Furthermore, mice and rats can be used to verify results for drug screening in lower animals in preclinical studies. Future work should focus on the identification of pathways in addition to Notch that are affected by loss of fucosylation and could potentially be targeted for therapeutic intervention. Our team is also currently investigating other fucosylation disruption models in Zebrafish, such as in GDP-fucose transfer, with the hope of understanding therapeutic options for patients with mutations in GDP-fucose transferases who are not responsive to oral fucose supplementation.

## Figures and Tables

**Table 1:** Summary of Fucosylation Pathways Gene Identified in Congenital Diseases of fucosylation or associated with complex diseases for which there are animal models discussed in this work.

Human Gene	Gene Function	Human Phenotype	Animal/Cell Mutant Allele	Animal/Cell Based Phenotype	References
*SLC35C1*	GDP-fucose transporter	LADII, leukocytosis, recurrent infections, growth retardation	*Slc35c1*^-/-^ (mouse) *nac*^-/-^ (*Drosophila*)	Growth retardation, reduced neutrophil rolling, hypocellular lymph nodes, dilated alveoles	[2,22,59,60,61]
*FCSK*	L-fucose kinase in salvage pathway	Severe infantile-onset epilepsy, neurodevelopmental delay, optical abnormalities	*fcsk*^-/-^ (zebrafish), *fcsk* morpholino	Seizures, cerebral hemorrhage, growth retardation, social behaviors, brain atrophy	[22,23,62]
*FUT8*	Core alpha 1,6-fucosyltransferase	Epilepsy, microcephaly, emphysema, myogenesis defect, congenital glaucoma	*Fut8*^-/-^ (mouse), *fut8* morpholino (zebrafish)	Growth retardation, reduced survival, abnormal lung development	[22,25,26,63,64]
*GFUS (FX)*	GDP-keto-6-deoxy mannose epimerase/reductase	Developmental delay, leukocytosis, colitis, adenocarcinoma, brain abnormalities, feeding aversion	*Gfus*^-/-^ (mouse)	Reduced survival, leukocytosis, colitis, adenocarcinoma	[22,27,65,66,67]
*POFUT1*	Protein O-fucosyltransferase	Dowling-Degos disease, liver fibrosis, hypopigmentation	*Pofut1* conditional KO (mouse), *pofut1* morpholino (zebrafish), *nti*^-/-^ (*Drosophila*)	Injury induced liver fibrosis, hypopigmentation	[32,33,34,68]
*GMDS*	GDP-mannose 4,6-dehydratase	congenital heart defect with Ebstein Anomaly, Glaucoma, cancer biomarker, cerebral small vessel diseases (CSVD)	*gmds*^-/-^ (zebrafish), *GMDS*-shRNA (cell lines), *gmd*^-/-^ mutant (drosophila)	Altered neural migration, synaptogenesis, hemorrhage, curly tail, reduced retinal ganglion cell number	[19,35,49,69,70,71,72]

## Data Availability

No new data were created or analyzed in this study.

## Abbreviations

GDP	Guanosine Diphosphate
GMDS	GDP-mannose 4,6-dehydratase
EGF	Epidermal Growth Factor
TSRs	Thrombospondin type
FUT	Fucosyltransferases
EMI	Elastin Microfibril Interface
HCC	Hepatocellular Carcinoma
TGF	Transforming Growth Factor
MMP	Matrix metalloproteinases
NICD	Notch Intracellular Domain
CDG	Congenital Disease of Glycosylation
GWAS	Genome Wide Association Studies
LADII	Leukocyte adhesion deficiency 2
CDG IIC	Congenital disorder of glycosylation type IIc
FPGT	fucose-1-phosphate guanylyl transferase
ER	Endoplasmic reticulum
POFUT	Protein O-Fucosyltransferase
POAG	Primary Open Angle Glaucoma
OMIM	Online Mendelian Inheritance in Man
FCSK	Fucokinase
GFUS	GDP-L-Fucose Synthase
DDD	Dowling-Degos disease
SNPs	Single nucleotide polymorphisms
CADASIL	Cerebral Autosomal Dominant Arteriopathy with Subcortical Infarcts and Leukoencephalopathy
CSVD	cerebral small vessel diseases
CS	congenital scoliosis
SCD	spondylocostal dysostoses
LNFG	Lunatic Fringe
PAR	passive avoidance memorization
RGCs	Retinal ganglion cells
OCT	optical coherence tomography
PTZ	Pentylenetetrazol
hpf	hour post fertilization
CEFT	*C. elegans* fucosyltransferases
